# ZEB2 stably represses *RAB25* expression through epigenetic regulation by SIRT1 and DNMTs during epithelial-to-mesenchymal transition

**DOI:** 10.1186/s13072-018-0239-4

**Published:** 2018-11-16

**Authors:** Nicolas Skrypek, Kenneth Bruneel, Cindy Vandewalle, Eva De Smedt, Bieke Soen, Nele Loret, Joachim Taminau, Steven Goossens, Niels Vandamme, Geert Berx

**Affiliations:** 1 Molecular and Cellular Oncology Laboratory, Department of Biomedical Molecular Biology, Ghent University, Technologiepark 927, 9052 Zwijnaarde, Ghent, Belgium; 2 Cancer Research Institute Ghent (CRIG), Ghent, Belgium; 3 Centre for Medical Genetics, Ghent University and University Hospital, Ghent, Belgium; 4 Data Mining and Modeling for Biomedicine, VIB Inflammation Research Center, Ghent, Belgium; 5 VIB-UGent Center for Inflammation Research, Technologiepark 927, 9052 Ghent, Belgium

**Keywords:** EMT, ZEB2, Epigenetic regulation, RAB25, SIRT1, DNMT

## Abstract

**Background:**

Epithelial mesenchymal transition (EMT) is tightly regulated by a network of transcription factors (EMT-TFs). Among them is the nuclear factor ZEB2, a member of the zinc-finger E-box binding homeobox family. ZEB2 nuclear localization has been identified in several cancer types, and its overexpression is correlated with the malignant progression. ZEB2 transcriptionally represses epithelial genes, such as E-cadherin (*CDH1*), by directly binding to the promoter of the genes it regulates and activating mesenchymal genes by a mechanism in which there is no full agreement. Recent studies showed that EMT-TFs interact with epigenetic regulatory enzymes that alter the epigenome, thereby providing another level of control. The role of epigenetic regulation on ZEB2 function is not well understood. In this study, we aimed to characterize the epigenetic effect of ZEB2 repressive function on the regulation of a small Rab GTPase RAB25.

**Results:**

Using cellular models with conditional ZEB2 expression, we show a clear transcriptional repression of *RAB25* and *CDH1*. RAB25 contributes to the partial suppression of ZEB2-mediated cell migration. Furthermore, a highly significant reverse correlation between *RAB25* and *ZEB2* expression in several human cancer types could be identified. Mechanistically, ZEB2 binds specifically to E-box sequences on the *RAB25* promoter. ZEB2 binding is associated with the local increase in DNA methylation requiring DNA methyltransferases as well as histone deacetylation (H3K9Ac) depending on the activity of SIRT1. Surprisingly, SIRT1 and DNMTs did not interact directly with ZEB2, and while SIRT1 inhibition decreased the stability of long-term repression, it did not prevent down-regulation of *RAB25* and *CDH1* by ZEB2.

**Conclusions:**

ZEB2 expression is resulting in drastic changes at the chromatin level with both clear DNA hypermethylation and histone modifications. Here, we revealed that SIRT1-mediated H3K9 deacetylation helps to maintain gene repression but is not required for the direct ZEB2 repressive function. Targeting epigenetic enzymes to prevent EMT is an appealing approach to limit cancer dissemination, but inhibiting SIRT1 activity alone might have limited effect and will require drug combination to efficiently prevent EMT.

**Electronic supplementary material:**

The online version of this article (10.1186/s13072-018-0239-4) contains supplementary material, which is available to authorized users.

## Introduction

Epithelial-to-mesenchymal transition (EMT) is an important reversible process that occurs during embryonic development and in physiological processes during adulthood (e.g., wound healing), but it is aberrantly activated in pathologies such as fibrosis and cancer progression. During EMT, epithelial cells lose cell polarity and acquire a more spindle-shaped mesenchymal morphology associated with transcriptional repression of epithelial genes such as E-cadherin (*CDH1*) and activation of mesenchymal genes such as Vimentin (*VIM*). In tumors, cells undergoing EMT become motile, promoting tumor invasion and metastasis, as well as stemness and chemoresistance, which make them more aggressive and able to drive tumor relapse [1, 2].

EMT is a tightly regulated process controlled by a network of transcription factors (EMT-TFs), including ZEB2, a member of the zinc-finger E-box binding homeobox (ZEB) family. ZEB2 is overexpressed in several cancer types (e.g., breast, ovarian and colorectal cancer) [3] which are correlated with metastasis and poor prognosis [4–7]. Like other EMT-TFs, ZEB2 represses epithelial genes by directly binding to E-box sequences in the promoter of its targeted genes or by activating mesenchymal genes through a mechanism that is still debated. Over the past few years, an increasing number of studies have shown that EMT-TFs can interact with many epigenetic remodeling enzymes to alter the epigenome of cells resulting in another level of gene regulation by EMT-TFs [8]. So far, only LSD1, a histone demethylase [9], and G9A, a histone methyltransferase [10], have been reported to interact with ZEB2. The existence of other epigenetic partners is suspected, as like in the case of other EMT-TFs. However, the importance of such nuclear interactions on the repressing/activating functions of EMT-TFs in gene regulation during EMT is still not clear.

While *CDH1* is a well-known target of EMT-TFs, many other genes are regulated during EMT but their regulation and effect on EMT-associated properties are not well understood. RAB25 (Rab11c), a small Rab GTPase belonging to the Rab11 family, is down-regulated during EMT [11, 12]. Physiologically, RAB25 is specifically expressed in epithelial cells and is involved in the intracellular trafficking associated with apical recycling and transcytosis [13]. Independent of EMT, RAB25 has gained attention because its expression is altered in different human cancer subtypes, but its role in cancer progression is not clear. Nevertheless, RAB25 seems to be tumor specific. Several studies on ovarian cancer [14], renal cell carcinoma [15, 16], luminal B breast cancer [12] and advanced non-small lung cancer [17] have described RAB25 as an oncogene, associated with metastasis and a poor prognosis. However, other studies have shown that RAB25 is a tumor suppressor that prevents cell migration and proliferation in head and neck squamous carcinoma [18], colorectal cancer [19, 20] and claudin-low breast cancer [12]. In EMT, RAB25 has repeatedly been found inversely correlated with EMT-TF [12, 21], suggesting that it has a more conserved functional role in epithelial differentiation. But little is known about the involvement and regulation of RAB25 during EMT.

In this study, we examined RAB25 as a new putative EMT regulator during ZEB2-induced EMT and studied the transcriptional regulatory mechanism by focusing on epigenetic changes.

## Results

### *RAB25* expression is inversely correlated with *ZEB2* expression in several cancer types

**Fig. 1:**
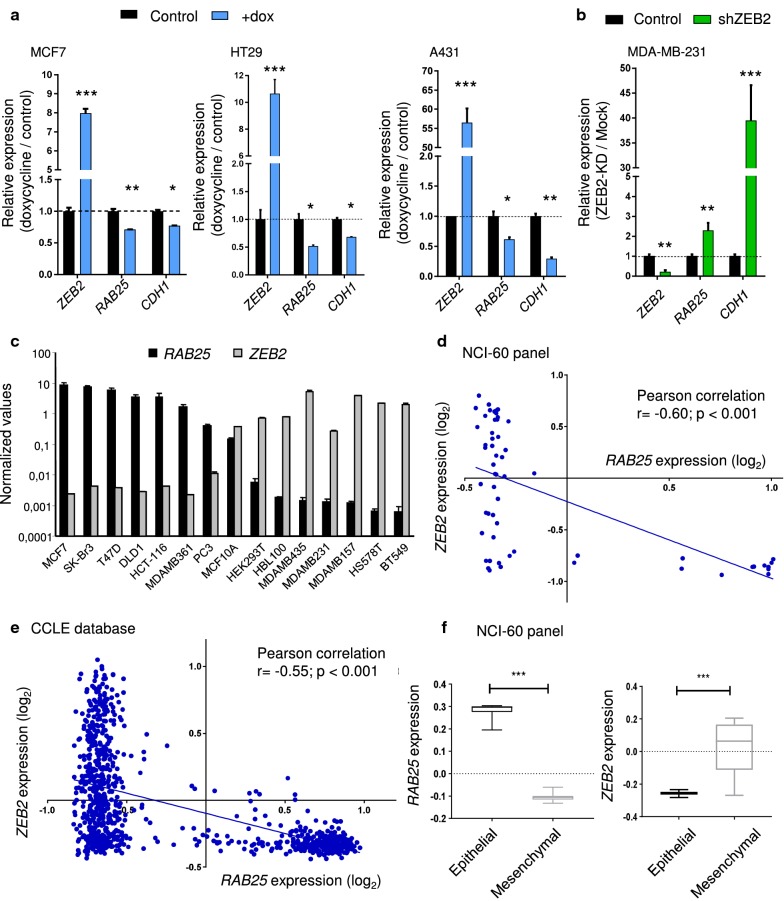
*ZEB2* and *RAB25* expression are inversely correlated. (**a**, **b**) Relative mRNA expression of *ZEB2*, *RAB25* and *CDH1* measured by qRT-PCR in (**a**) MCF7, HT29 and A431 ZEB2 doxycycline-inducible (+dox) models and **b** MDA-MB-231 ZEB2 KD (shZEB2). Control values were set to 1, and s.d. is shown. *P* values were determined using unpaired *t* tests (*p < 0.05;**p < 0.01). Three independent experiments were performed. **c** *ZEB2* and *RAB25* mRNA expression in a epithelial cell line panel. **d**–**e** Correlation between *ZEB2* (*y*-axis) and *RAB25* (*x*-axis) expression from publicly available datasets from **d** NCI-60 cell panel and **e** the Cancer Cell Line Encyclopedia (CCLE). Mean of each parameter was calculated, individual cell type values reported to the mean and log2 transformed. Pearson’s correlation test was used to calculate *r* and *p* values. **f** Boxplots showing *RAB25* (left panel) and *ZEB2* (right panel) expression in epithelial (*n* = 11) and mesenchymal (*n* = 37) cell lines from NCI-60 cell panel. *P* values were determined using unpaired *t* tests (***p < 0.001)

In previous studies, we and others have shown by genome-wide expression analysis that induction of EMT-TFs down-regulates *RAB25* expression (data not shown; [12, 21]). In this study, we specifically examined the effect of ZEB2 on tumor cell properties associated with differential gene expression. To that end, we generated a doxycycline-inducible ZEB2 construct stably transduced into MCF7, A431 and HT29 cell lines which have low or no endogenous ZEB2 expression. We found that *ZEB2* expression correlated with a down-regulation of both *RAB25* and *CDH1* (Fig. 1a) coding for E-cadherin and a well-known target of ZEB2 [22]. This confirms the relevance of our model for EMT at the molecular level. Knocking-down ZEB2 (ZEB2-KD) in the mesenchymal-like MDA-MB-231 breast cancer cell line (which expresses more endogenous ZEB2 and less RAB25 than MCF7 cells) induced the expression of *RAB25* and *CDH1* (Fig. 1b). In RNA from a panel of cell lines of epithelial origin, we found a significant reverse correlation between *ZEB2* and *RAB25* expression levels (Fig. 1c). To evaluate if this correlation is broadly represented in other cancer types, we analyzed the NCI-60 cell line transcriptome database and the Cancer Cell Line Encyclopedia (CCLE). Indeed we confirmed that *RAB25* expression is inversely correlated with *ZEB2* expression (*r* = − 0. 60; *p* < 0.001 and *r* = − 0.55; *p* < 0.001 in the two respective databases) indicating the existence of a conserved regulatory mechanism (Fig. 1d, e).

**Fig. 2:**
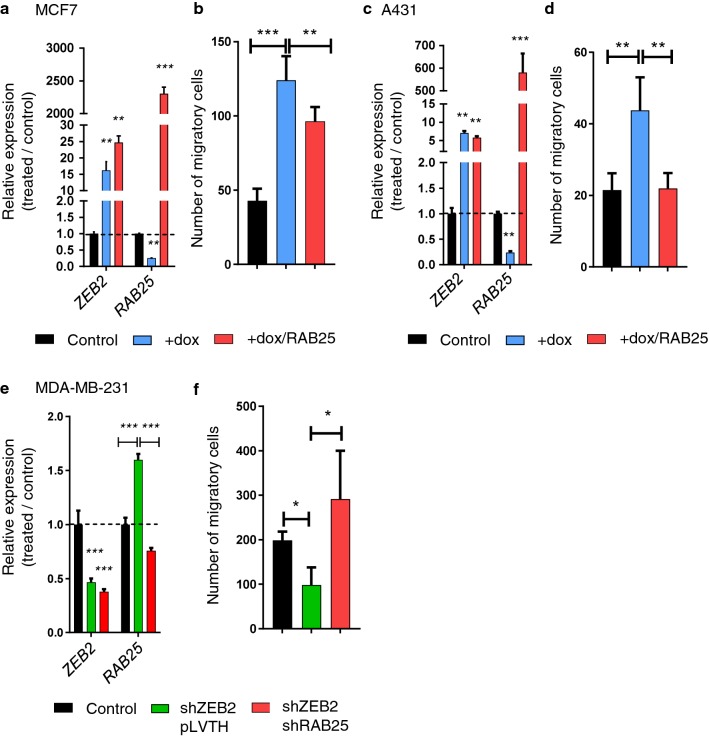
*RAB25* expression reduced ZEB2-driven cell migration. **a**–**d** ZEB2 was induced in MCF7 and A431 (+dox), and RAB25 expression was rescued by a transiently transfected RAB25 expression construct (+dox/RAB25). **a**, **c** mRNA expression level of *ZEB2* and *RAB25* was analyzed by qRT-PCR in (**a**) MCF7 and **c** A431. Control values were set to 1, and s.d. is shown. **b**, **d** Cell migration was evaluated by a transwell assay with **b** MCF7 and **d** A431. Results are expressed as the total number of cells counted per chamber. **e**–**f** ZEB2 was knocked down in MDA-MB-231 (shZEB2/pLVTH), and RAB25 induction was blocked using specific shRNA (shZEB2/shRAB25). **e** mRNA expression level of *ZEB2* and *RAB25* was analyzed by qRT-PCR. Control values were set to 1, and s.d. is shown. **f** Cell migration was evaluated by a transwell assay. Results are expressed as the total number of cells counted per chamber. For all analyses, *p* values were determined using one-way ANOVA (*p < 0.05; **p < 0.01; ***p < 0.001). Values represent the means of three independent experiments

The correlation between *RAB25* and *ZEB2* expression was strong in breast cancer and colorectal cancer cells (respectively, *r* = − 0.84; *p* < 0.001 and *r* = − 0.66; *p* < 0.001, Additional file 1: Fig. S1a and c), but low in pancreatic and small-cell lung cancer cells (respectively, *r* = − 0.39; *p* < 0.001 and *r* = − 0.38; *p* < 0.001, Additional file 1: Fig. S1d and e). Interestingly, most breast cancer subtypes expressed a high level of *ZEB2* and low level of *RAB25*, except in claudin-low subtype in which RAB25 has been described as a tumor suppressor (Additional file 1: Fig. S1b). Almost all skin cancer cell lines had high expression levels of *ZEB2* and low levels of *RAB25* (Additional file 1: Fig. S1f). In non-small-cell lung cancer cells, *ZEB2* expression was weak regardless of the *RAB25* expression level (Additional file 1: Fig. S1g). Grouping cell lines from the NCI-60 panel on the basis of their epithelial (*n* = 11) or mesenchymal-like (*n* = 37) differentiation showed that *RAB25* was essentially expressed in epithelial cells, while *ZEB2* was expressed in mesenchymal cells (Fig. 1e). Taken together, these data show that RAB25 is likely important in epithelial polarity and is potentially transcriptionally repressed by ZEB2 during EMT. To study the functional effect of RAB25 attenuation in the context of ZEB2-induced EMT, we performed RAB25 rescue experiments. To that end, we conditionally induced ZEB2 in MCF7 and A431 cell lines and then overexpressed RAB25 to counteract its repression by ZEB2 (Fig. 2a, c). Upon ZEB2 induction, cell migration was significantly increased (Fig. 2b, d) while the rescue of RAB25 expression partially prevented cell migration (Fig. 2b, d). Using MDA-MB-231 ZEB2-KD cells, we performed the reverse procedure by blocking RAB25 induction upon ZEB2 down-regulation using specific shRNAs (Fig. 2e). We found a significant decrease in cell migration, while RAB25 KD along with ZEB2 KD drastically increased migration (Fig. 2f). Together, these data support the notion that RAB25 represses ZEB2-associated cell migration during EMT.

### ZEB2 directly binds to E-box sequences in the RAB25 promoter

**Fig. 3:**
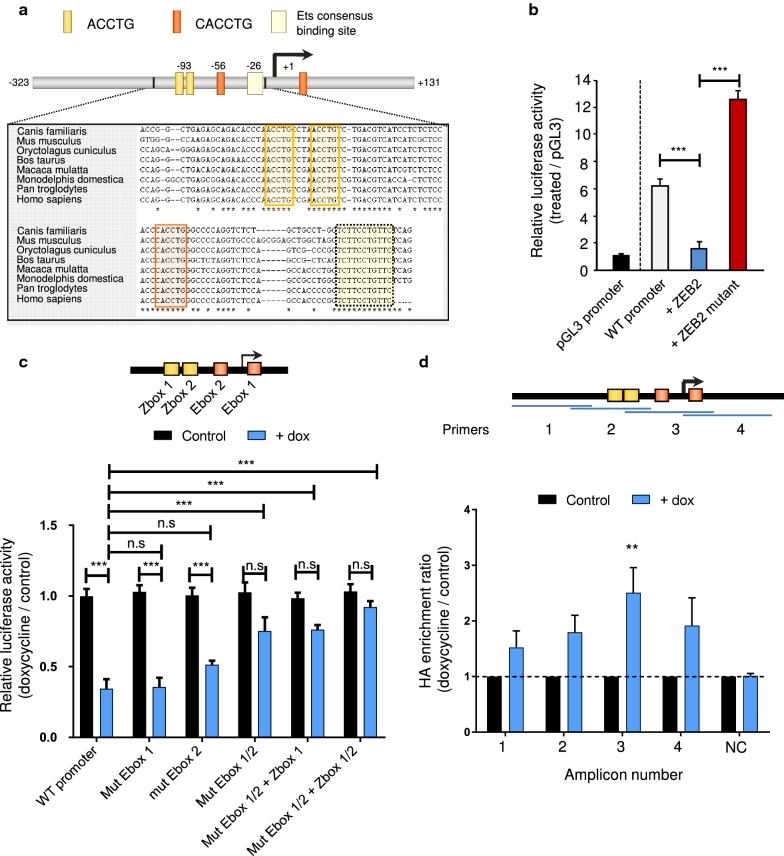
ZEB2 binds the *RAB25* promoter and represses its transcriptional activity. **a** Scheme representing *RAB25* promoter sequence with E- (CANNTG) and Z-boxes (ATANNTGT). **b** Luciferase activity of *RAB25* promoter was measured 24 h after transfection with or without ZEB2-WT or ZEB2 double DNA-binding mutant. pGL3 promoter activity was used as control and set as 1, and s.d is shown. **c** A simplified schematic of *RAB25* promoter showing localization of E/Z-boxes. Luciferase activity of *RAB25* promoter WT and mutated for E/Z-boxes was measured after ZEB2 induction (+dox). Control values were set as 1, and s.d. is shown. **d** HA-ZEB2 ChIP assay analyzed at different locations in the *RAB25* promoter. Amplicons number and location are depicted on a simplified schematic of the *RAB25* promoter. Enrichments to input were calculated, control values were set as 1 and s.d.is shown. *NC* negative control. For all analyses, *P* values were determined using two-way ANOVA (**p < 0.01; ***p < 0.001; *ns* nonsignificant). Three independent experiments were performed for all experiments

The regulation of *RAB25* expression by ZEB2 is not well understood, so we studied its transcriptional control. Based on the results of our previous study, we analyzed the promoter region of *RAB25* from −200 bp to + 17 bp. We identified putative ZEB2 binding sites in the *RAB25* promoter: two E-box sequences (CANNTC) and two Z-box sequences (ATANNTGT), which are conserved in several species (Fig. 3a). To study the impact of ZEB2 on RAB25 promoter activity, we cloned the identified promoter region in a luciferase reporter vector. Upon ZEB2 induction, *RAB25* promoter activity was greatly reduced in RAB25-positive cells, while the overexpression of ZEB2 harboring mutations in both zinc-finger clusters responsible for DNA binding failed to repress the activity (Fig. 3b). This result shows that ZEB2 binding to DNA is essential for control of the *RAB25* promoter activity. To examine if the identified E- and/or Z-boxes are involved in ZEB2 recruitment and to identify which of them are essential, we mutated the sites individually and also in different combinations. Mutation of E-box 1 or E-box 2 alone did not significantly affect RAB25 repression, whereas mutation of both of them diminished RAB25 repression substantially (Fig. 3c). Combining Z-box 1 and 2 mutations completely reversed the repression (Fig. 3c). To confirm at the chromatin level the precise binding region of ZEB2, we performed ZEB2 chromatin immunoprecipitation (ChIP) assays using PCR amplicons covering the entire *RAB25* promoter (Fig. 3d). We found that ZEB2 was significantly enriched in amplicon 3, which corresponds to E-box 2 and E-box 1 localization (Fig. 3d). We analyzed ZEB2 enrichment at the *CDH1* and *EpCAM* promoters as positive controls because those genes had been reported to be targeted by ZEB factors [11, 23]. As expected, we confirmed the enrichment of ZEB2 for both genes (Additional file 2: Fig. S2a). Taken together, our results indicate that ZEB2 directly interacts with E-box sequences in the RAB25 promoter to repress transcription.

### ZEB2 increased DNA methylation at the RAB25 promoter through DNMTs activity

**Fig. 4:**
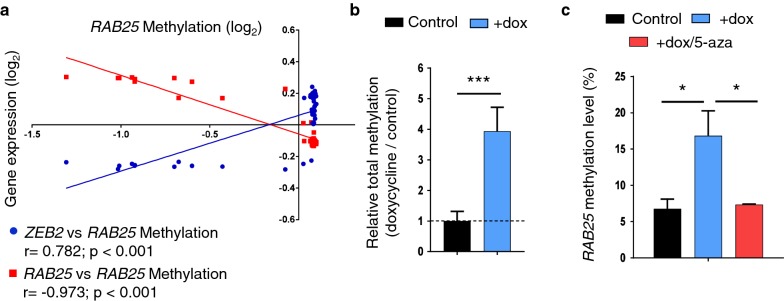
ZEB2 increased DNA methylation at *RAB25* promoter through DNMTs activity. **a** Correlation between *RAB25* DNA methylation (*x*-axis) and *ZEB2* (blue dots) or *RAB25* (red dots) expression (*y*-axis) from publicly available NCI-60 cell panel datasets. Mean of each parameter was calculated, individual cell type values reported to the mean and log2 transformed. Pearson’s correlation test was used to calculate *r* and *P* values. **b** Global DNA methylation level upon ZEB2 induction (+dox) in MCF7 was measured by ELISA assay. Control values were set to 1, and s.d. is shown. *P* values were determined using unpaired *t* tests (***p < 0.001). **c** RAB25 promoter DNA methylation in MCF7 was measured by methyl-binding domain (MBD) assay after ZEB2 induction (+dox) and 5′-aza-2-deoxycytidine (+dox/5-aza) treatment. Enrichments to input were calculated, control values were set as 1 and s.d. is shown. *P* values were determined using one-way ANOVA (*p < 0.05). Three independent experiments were performed

An increasing number of studies document the interaction of different EMT-TFs with different combinations of epigenetic enzymes and show that the genomes of cells going through EMT undergo large shifts in their epigenomes. However, the functional contribution of these epigenetic alterations during EMT is still under investigation. To focus on the first epigenetic modifications directly caused by ZEB2 activity, we measured DNA methylation and different histone marks 24 h after ZEB2 induction in epithelial cells. Low *RAB25* expression has been linked with a high DNA methylation level [24–27]. We analyzed the *RAB25* methylation status using the methylome dataset of the NCI-60 cell panel (GSE49143) focusing on cell lines previously shown to have a strong inverse correlation between *ZEB2* and *RAB25* (Fig. 1d; 41 out of 60 cell lines). For three out of four probes, we found that *ZEB2* expression was directly correlated with a high DNA methylation level at the *RAB25* promoter (*r* = 0.782; *p* < 0.001), while *RAB25* expression was inversely correlated with the methylation status (*r* = − 0.973; *p* < 0.001) (Fig. 4a). In our model, ZEB2 induction leads to a strong increase in the DNA methylation (Fig. 4b) To specifically measure *RAB25* DNA methylation controlled by ZEB2, we performed a methyl-binding protein assay and analyzed the *RAB25* promoter region. There was a significant increase in *RAB25* methylation in the presence of ZEB2 expression, while 5-aza-2′-deoxycytidine (5-aza) treatment prevented methylation (Fig. 4c). This result points to a direct role for ZEB2 in *RAB25* promoter methylation by DNA methyltransferases.

### ZEB2 increased H3K9 deacetylation at the *RAB25*, *CDH1* and *EpCAM* promoters through SIRT1 activity

**Fig. 5:**
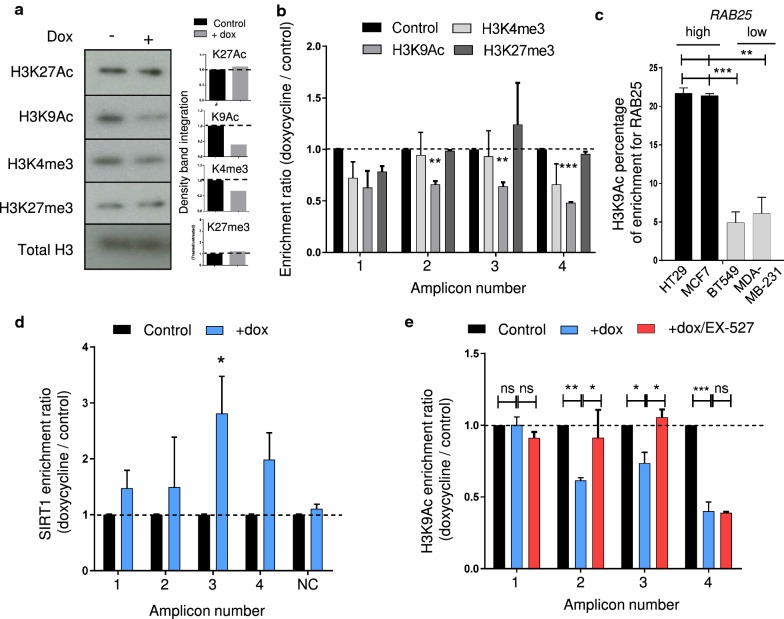
ZEB2 increased H3K9Ac deacetylation at *RAB25* promoter through SIRT1 activity. **a** Immunoblotting of histone mark upon ZEB2 induction (+dox) in MCF7. The density of each marker was measured and represented as histograms. Expression in control was set to 1. **b** Histone ChIP assay analyzed at different localizations on *RAB25* promoter after ZEB2 induction (+dox) in MCF7. **c** H3K9Ac ChIP assay performed in high (black) and low (gray) *RAB25* expressing cell lines. **d** SIRT1 ChIP assay analyzed at different localizations on *RAB25* promoter after ZEB2 induction (+dox) in MCF7. **e** H3K9Ac ChIP assay was performed after ZEB2 induction (+dox) with SIRT1 inhibitor (EX-527, 1 µM) (+dox/EX-527) in MCF7 and analyzed at different localizations on *RAB25* promoter. For all ChIP analyses, enrichments to input were calculated, control values were set as 1 and s.d. is shown. *NC* negative control. *P* values were determined using two-way ANOVA (*p < 0.05; **p < 0.01; ***p < 0.001). Three independent experiments were performed

Additionally, we wanted to extend our knowledge of the epigenetic regulation of *RAB25* by measuring the level of several histone marks related to transcriptional activation (H3K27Ac, H3K4me3 and H3K9Ac) or repression (H3K27me3). After ZEB2 induction, H3K4me3 and H3K9Ac histone marks decreased substantially, but H3K27Ac and H3K27me3 were not affected (Fig. 5a). We performed histone ChIP assay and measured the enrichment of H3K9Ac, H3K27me3 and H3K4me3 at the *RAB25* promoter using the same PCR amplicon as in Fig. 3d, to examine the local effect of ZEB2 on the chromatin status (Fig. 5b). Upon ZEB2 induction, H3K9Ac decreased significantly across the RAB25 promoter but H3K4me3 and H3K27me3 were not significantly altered (Fig. 5b). Additionally, knockdown of ZEB2 in MDA-MB-231 cells significantly increased H3K9Ac, confirming a link between ZEB2 and H3K9Ac (Additional file 2: Fig. S2b).

We compared the basal levels of H3K9Ac at the *RAB25* promoter in a series of cell lines with differential *RAB25* expression levels (RAB25 + : MCF7 and HT29; RAB25-: MDA-MB-231 and BT549, Additional file 2: Fig. S2c). *RAB25*-high cells have high H3K9Ac levels, and *RAB25*-low cells have low levels (Fig. 5c). To understand how ZEB2 affects the H3K9Ac histone modification, we focused our attention to histone deacetylases that are not affecting H3K27Ac but specifically regulating H3K9Ac. The available literature pointed us toward sirtuin family with SIRT1, which has been shown to strongly deacetylate H3K9Ac without affecting H3K27Ac [28–30]. By performing a SIRT1 ChIP assay, we showed that SIRT1 is significantly enriched upon ZEB2 induction (Fig. 5d), and correlated with ZEB2 recruitment (Fig. 3d). To see whether SIRT1 affects H3K9Ac, we treated ZEB2-induced cells with a SIRT1-specific inhibitor (EX-527, 1 µM). This inhibitor globally prevented the H3K9Ac decrease caused by ZEB2, confirming a role for SIRT1 in H3K9 deacetylation (Fig. 5e). We extended these results to the *EpCAM* gene showing an enrichment of SIRT1 correlated with H3K9Ac (Additional file 2: Fig. S2d and e). The same was observed for *CDH1* with H3K9Ac shift blocked by the SIRT1 inhibitor (Additional file 2: Fig. S2e), but SIRT1 was not significantly enriched (Additional file 2: Fig. S2d) suggesting that SIRT1 binds to another location on the *CDH1* promoter. Altogether, these results indicate that during ZEB2-induced EMT, SIRT1/H3K9Ac and DNMT/DNA methylation participate in a mechanism of repressing the expression of *RAB25* mRNA.

### SIRT1 activity maintains the stability of ZEB2-induced RAB25 repression

**Fig. 6:**
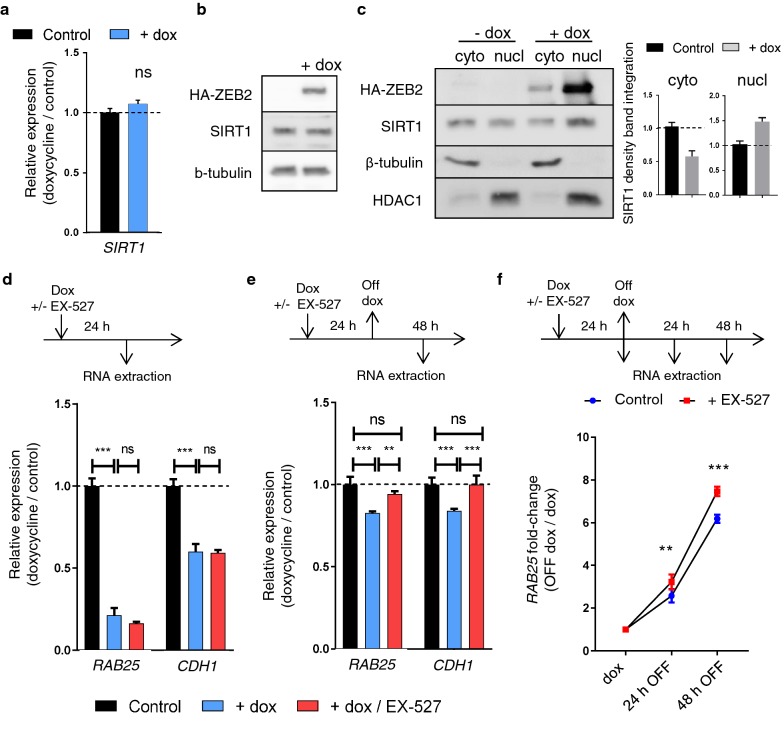
SIRT1 nuclear activity maintains *RAB25* repression stability. **a**–**c** Expression and localization of ZEB2 and SIRT1 in MCF7 upon ZEB2 induction (+dox) measured by **a** qRT-PCR and immunoblotting from **b** total protein extracts or **c** cytoplasmic (cyto) and nuclear (nucl) extracts. SIRT1 band density was measured and represented as histograms. Expression in control was set to 1. **d**, **e** *RAB25* and *CDH1* mRNA expression were measured by qRT-PCR **d** 24 h after ZEB2 induction (+dox) with SIRT1 inhibitor (EX-527, 1 µM) (+dox/EX-527) or **e** 24 h after ZEB2 induction and 48 h of doxycycline withdrawal (+dox) with SIRT1 inhibitor (EX-527, 1 µM) (+dox/EX-527). **f** Kinetic of *RAB25* expression measured by qRT-PCR 24 h after ZEB2 induction and 24 or 48 h after doxycycline withdrawal (control) with SIRT1 inhibition (EX-527, 1 µM) (+EX-527). *RAB25* level at control time point was set to 1, and s.d is shown. For all analyses, *p* values were determined using two-way ANOVA (**p < 0.01;***p < 0.001; *ns* nonsignificant). Three independent experiments were performed

Based on the observed associations of ZEB2, DNA methylation/DNMTs and H3K9Ac/SIRT1 with the *RAB25* promoter, we hypothesized the existence of a direct interaction between those nuclear proteins. Therefore, we performed a co-immunoprecipitation assay to pull down ZEB2. However, we could not pull down SIRT1 or DNMTs (data not shown), suggesting that SIRT1 and DNMTs are recruited indirectly to *RAB25*. As SIRT1 expression is not altered upon ZEB2 induction, regulation of SIRT1 expression by ZEB2 can be ruled out (Fig. 6a, b). Interestingly, we observed that compared to control cells, SIRT1 decreased in the cytoplasmic fraction but increased in the nuclear fraction in response to ZEB2 induction (Fig. 6c). This suggests that ZEB2 increased SIRT1 activity through an indirect mechanism by increasing its localization in the nucleus.

Finally, to understand the functional effect of the ZEB2-SIRT1-H3K9Ac association on gene repression, we examined *RAB25* and *CDH1* expression after SIRT1 inhibition. As shown previously, upregulating ZEB2 down-regulated *RAB25* and *CDH1*. However, SIRT1 inhibition is not sufficient to prevent repression of *RAB25* and *CDH1* (Fig. 6d) even when H3K9Ac protection was demonstrated (Fig. 5e). We next hypothesized that deacetylation of H3K9 might stabilize transcriptional repression. Therefore, we induced *ZEB2* in order to repress *RAB25* and removed doxycycline, which quickly stops *ZEB2* transcription. After 48 h, the levels of *RAB25* and *CDH1* were higher when SIRT1 was inhibited compared to the normal condition (Fig. 6e). We repeated the experiment and examined two time points (24 h and 48 h). We found that the increase in *RAB25* and *CDH1* was significantly more rapid when SIRT1 was inhibited in comparison to control conditions (Fig. 6f). However, by repeating the experiment with the DNMTs inhibitor 5-aza, with or without the SIRT1 inhibitor, we showed no synergic effect but, surprisingly, decreased recovery of *RAB25* expression (Additional file 3: Fig. S3a). One possible explanation is that the wider effect of 5-aza on gene regulation increased the expression of EMT-TFs such as *ZEB1*, *SNAI1* and *SNAI2* (Additional file 3: Fig. S3b), which in turn repressed *RAB25* when EMT-TFs were overexpressed individually (Additional file 3: Fig. S3c).

## Discussion

We show for the first time that ZEB2 induction leads to a strong repression of *RAB25* involving an epigenetic mechanism encompassing DNA methylation and histone modification to stabilize gene repression.

Most studies on gene regulation during EMT have focused on *CDH1,* but other EMT-targeted genes have received inadequate attention. RAB25 is a small GTPase that is specifically expressed in epithelial cells [21] as confirmed in our analysis and down-regulated during EMT [11, 12]. *RAB25* expression is altered in many cancer types (e.g., breast, colorectal, ovarian and lung). Intriguingly, it has been described as having both pro- and anti-tumorigenic properties, influencing proliferation and cell migration [12, 14, 16–20]. We observed RAB25 opposite function in our cell lines as specific knockdown of RAB25 altered migratory properties of A431 cells but had no effect on MCF7 cells (data not shown). However, in the EMT context the role of RAB25 seems to be more consistent. In ZEB2-induced EMT, *RAB25* down-regulation facilitates migration of cell lines from different origins, such as luminal-like (MCF7) or claudin-low (MDA-MB-231) breast cancer cells, and skin squamous carcinoma (A431) or colorectal cancer cells (HT29). It would be interesting to know whether the RAB25 migratory effect also applies to other EMT-TFs, because they are usually co-expressed and work in concert. The pro-migratory effect of RAB25 observed in some cellular models is more dependent on the cellular context for the expression of specific RAB25-partners as CLIC3 [31]. However, in tumors which contain a mosaic of cells with different phenotypes, EMT-mediated and specific RAB25-mediated migration could work in concert, with EMT-positive cells at the edge of the tumor leading the way for *RAB25*-positive epithelial cells cooperating to promote tumor progression. Another functional effect of *RAB25* expression could be the induction of mesenchymal-to-epithelial transition (MET) at disseminated cancer cell secondary sites and promotion of colonization, as proposed by Mitra and colleagues [12].

Understanding EMT-regulatory mechanisms that repress genes such as *RAB25* could identify new therapeutic targets to reverse EMT-associated properties, for example, metastasis, stemness and chemoresistance. Re-expressing RAB25 might not only affect the migratory properties as mentioned earlier but could also lead to the sensitization of cancer cells to specific therapies. Due to the physiological role of RAB25 in apical recycling, it has been associated with speed of EGFR recycling [32], making cells more sensitive to EGFR-targeted therapies such as gefitinib [33]. However, targeting nuclear factors as EMT-TFs is difficult, and other strategies are needed to disrupt the EMT-regulatory network. An appealing alternative approach is to target essential EMT-TFs cofactors such as chromatin-remodeling enzymes to restore expression of *RAB25*.

The link between EMT and epigenetic regulation was described a decade ago [34, 35] and is increasingly pointed to an important aspect upstream and downstream of the EMT-regulatory network [8]. During EMT, the epigenome of cells is drastically altered by hypermethylation, associated with repressive histone marks (H3K27me3), of epithelial genes such as *CDH1* or *GRHL2*. In contrast, mesenchymal genes showed hypomethylation of the promoter and/or gene body hypermethylation associated with active histone marks (H3K4me3/H3K9Ac), as described for *TCF4* [11, 36–40]. Our study sheds light on the specific effect of ZEB2 on gene regulation. We showed that upon ZEB2 induction, there is considerable global hypermethylation and a shift of active to repressive histone marks, resulting in a general repressed chromatin. To try to understand this repressive shift, we turned our attention to the activities of DNMTs and SIRT1.

DNMTs have been linked with EMT-TFs function, and a direct interaction of DNMT1 with SNAI1/2 and ZEB1 factors has been demonstrated [41–43]. ZEB2 was previously shown to require DNMTs activity to function in embryonic stem cell differentiation [44], but there was no direct evidence for the interaction of ZEB2 with DNMTs. In our models, we found no evidence for a direct interaction between ZEB2 and DNMTs. The effect of DNA methylation on gene regulation during EMT has been reported mainly for *CDH1,* and there is some discrepancy between the studies [42, 45]. Fukagawa and collaborators showed in a panel of breast cancer cell lines that *CDH1* has different levels of methylation that correlate with ZEB1 and ZEB2 expression levels [42]. Treatment of cells having low *CDH1* expression with 5-aza is not enough to revert *CDH1* repression but requires that ZEB1 and ZEB2 have to be knocked down to strongly restore *CDH1* expression. Our study is consistent with these results, as 5-aza alone was not enough to restore *RAB25* and *CDH1* expression. In other studies, treatment with 5-aza was enough to revert *CDH1* repression, and it has been described as an anti-EMT drug [45]. In other studies, 5-aza treatment induced EMT [46]. From a therapeutic perspective, these conflicting findings show that 5-aza treatment is a double-edged sword. In some tumor types, it blocked EMT and tumor progression [46, 47], while in others induced EMT-TFs expression and EMT [45], like we observed in the induction of SNAI1, SNAI2 and ZEB1 expression in MCF7 cells after 5-aza treatment alone or in combination with the SIRT1 inhibitor.

The second significant effect of ZEB2 induction on epigenetic modification was the deacetylation of H3K9 by SIRT1 deacetylase. SIRT1 resides in the nucleus where it deacetylates histones (*e.g.,* H4K16 and H3K9) and non-histone proteins (e.g., KU70; p53…) [28, 29]). SIRT1 elevation has been associated with tumor progression and a worse prognosis in several cancer types (e.g., bladder, breast, hepatocellular carcinoma, gastric, and pancreatic cancer) [48–53]. In vitro and in vivo experiments linked the induction of SIRT1 to an increase in cell proliferative and migratory properties inversely correlated with *CDH1* expression [48, 54–56]. As suspected with *CDH1* down-regulation, SIRT1 pro-migratory property has been linked with EMT and has been an essential actor for the TGF-β-induced EMT [52, 53, 57–59]. Mechanistically, SIRT1 interacts with ZEB1 and MPP8 to repress *CDH1* gene expression [60, 61]. Our data show that compared to ZEB1, ZEB2 cannot interact with SIRT1 even if both of them are enriched at the same *RAB25* promoter region. We can exclude the regulation of SIRT1 by ZEB2 as we could not see differences in expression level but an increase in SIRT1 in the nucleus of cells expressing ZEB2. Several possible scenarios can explain the indirect effect of ZEB2 on SIRT1 and DNMTs actions, as (i) the modulation of signaling pathways affecting enzymes activity and localization (ii) the creation of early DNA marks recruiting epigenetic complex containing SIRT1 and DNMTs in a second wave or (iii) through the regulation of SIRT1 and DNMTs partners, promoting and/or dictating DNA interaction specificity.

Another surprising result in our model came from the use of 5-aza with or without SIRT1 inhibition, which reversed DNA methylation and histone deacetylation but did not prevent *RAB25* and *CDH1* repression. These observations with the fact that ZEB2 is not directly recruiting DNMTs and SIRT1 point to a secondary regulatory event, whereby epigenetic modifications strengthen long-term gene repression but are not essential for the ZEB2 repressive function. However, EMT-TFs function dependency against epigenetic enzyme activity might be important for another protein complex. Such is the case for ZEB2 and LSD1, where inhibiting LSD1 or blocking the interaction partially prevents ZEB2-induced EMT [9], and for SNAI1 and EZH2, where targeting the long non-coding HOTAIR prevents SNAI1-EZH2 interaction and hepatocyte transdifferentiation through EMT [62]. Epigenetic enzymes other than LSD1 could be more important for ZEB2 functions. Investigating this possibility requires deciphering the entire ZEB2 interactome, especially in the earliest steps of ZEB2 activation, to fully be able to disrupt ZEB2 repressive and activating functions.

## Materials and methods

### Cell culture and cell models

A431, HT29 and MDA-MB-231 cells were grown in Dulbecco’s modified Eagle’s medium containing 4.5 g/l glucose (Gibco, Thermo Fisher Scientific, Bleiswijk, the Netherlands) supplemented with 10% fetal bovine serum (Bodinco, Alkmaar, the Netherlands) and 2 mM l-glutamine (Lonza, Verviers, Belgium). MCF7 cells were grown in Dulbecco’s modified Eagle’s medium containing 4.5 g/l glucose (Gibco, Life Technologies) supplemented with 5% fetal bovine serum (Bodinco), 0.01 mg/ml human recombinant insulin, 2 mM l-glutamine (Lonza), 1 mM sodium pyruvate (Sigma-Aldrich, Overijse, Belgium), and 1X non-essential amino acids (Lonza). All media contained 100 U/ml penicillin/streptomycin (Gibco, Llife Technologies), and cells were grown at 37 °C in an incubator in a 5% CO_2_ atmosphere.

Cellular models with conditional ZEB2 expression were obtained following the stable transduction of MCF7, HT-29 and A431 cells with a pSIN vector encoding ZEB2 ORF linked to the hemagglutinin (HA) tag and also encoding green fluorescent protein (GFP) to select transduced cells by flow cytometry. Clones were isolated and selected based on ZEB2 induction level upon doxycycline treatment. MDA-MB-231 ZEB2-KD cells were obtained following the stable transduction of pLVTH vector encoding ZEB2 shRNA (GGAGCTGGGTATTGTTAAA) and also GFP. The empty vector (pLVTH) was used to generate control cells.

### Transwell migration assays

A transwell migration assay was performed in Boyden chamber 24 transwell plates (8 µm pores, Corning, Sigma-Aldrich). Two days before the experiment, the cells were transfected, by using FuGENE HD (Promega, Leiden, the Netherlands), according to the manufacturer’s instructions, with (1) pWPI vector encoding RAB25 ORF in the MCF7 and A431 ZEB2-inducible cell models or (2) pLVTH vector encoding a shRNA specifically targeting RAB25 (GGCCCGAATGTTCGCTGAA) in the MDA-MB-231 ZEB2-KD cell model. The empty vector (pWPI or pLVTH) was transfected in parallel and used as control. After 24 h, the media were removed and replaced with media containing 2% FBS with or without doxycycline. After another 24 h, 5 × 10^4^ cells were seeded in the top chamber, while the lower chamber contained full medium to serve as chemoattractant, and maintained for 24 h. The cells on the lower surface were fixed, stained with DAPI, and counted using a fluorescent microscope. Each experiment was performed at least three times.

### Chromatin immunoprecipitation (ChIP)

For histone marks, 1 × 10^6^ cells were used per ChIP, while 2.5 × 10^6^ cells were used for HA and SIRT1 ChIP. Briefly, the cells were cross-linked with 1% paraformaldehyde in fixation buffer (Active Motive, La Hulpe, Belgium). After the isolation of the nuclei, DNA was fragmented with 25 U micrococcal nuclease for 30 min at 37 °C in micrococcal nuclease-digesting buffer (50 mM Tris–HCl pH 7.6, 1 mM CaCl_2_ and 0.05% Triton X-100). DNA fragment size (150-500 bp) was confirmed in a 1.2% agarose gel. The fragmented chromatin was incubated overnight at 4 °C with 5 µg anti-HA (ab9110, Abam, Cambridge, UK) or anti-SIRT1 (07-131 EMD Millipore, Sigma-Aldrich) or for 6 h at 4 °C with 3 µg anti-H3K27me3 (39155), anti-H3K4me3 (39159) or anti-H3K9ac (39917) (Active Motive), followed by the pull-down of protein–DNA complexes with A/G-conjugated magnetic beads for 1 h (EMD Millipore, Sigma-Aldrich). After the cleaning steps, DNA was purified with iPure V2 kit following the manufacturer’s protocol (Diagenode, Liège, Belgium).

### DNA methylation ELISA and Methyl-Binding Domain (MBD) assay

DNA was isolated from the cells with the DNeasy Blood and Tissue kit (Qiagen, Venlo, the Netherlands). Global DNA methylation was measured by ELISA with the fluorometric MethylFlash Methylated DNA 5-mC quantification kit (Epigentek, Farmingdale, NY, USA) following manufacturer’s protocols. Specific *RAB25* promoter methylation was measured after DNA methylation pull-down using MethylCap kit (Diagenode). Briefly, 1ug of DNA was sheared to generate fragments around 400 bp and was confirmed on a 1.2% agarose gel. Sheared DNA was incubated for 2 h at 4 °C with MethylCap protein, which will specifically bind to methylated DNA, followed by the pull-down of protein-DNA complex with magnetic meDNA capture beads provided in the kit. After cleaning steps, DNA was removed from the beads with High Elution buffer and analyzed by qPCR.

### Promoter isolation and reporter assays

The human *RAB25* promoter sequence was identified by screening public human genomic DNA databases (http://genome.ucsc.edu and http://www.ensembl.org). This sequence was aligned to orthologous sequences of multiple species using Mulan (http://mulan.dcode.org), and evolutionarily conserved transcription factor binding sites were identified. The human promoter sequence was amplified by PCR from genomic DNA (primers: 5′-GTGCTGGGATTACAGGCGTGAG-3′ and 5′-CTGGTCCTGCCCCTCCTCTCAT-3′. The 454-bp amplicon was first cloned in pCR-BluntII-TOPO (Thermo Fisher Scientific) and then subcloned in pGL3basic (Promega) using KpnI and XhoI. Mutagenesis of the putative ZEB2-binding sites in the human *RAB25* promoter sequence was performed with the QuickChange Multi Site-Directed Mutagenesis Kit (Agilent Technologies, Machelen, Belgium) using three primers, each mutated in the putative ZEB2-binding sequence: (1) mutant primer E-box 1 (CACCTG sequence): 5′-ATCTCTCCACCCATCTGGGCCCCAGGTCT C-3′;(2) mutant primer E-box 2 (CACCTG sequence): 5′-TTACAGCACCCCCATCTGCCAGAGCTGATC-3′; (3) and mutant primer Z-box1/2 (ACCTG sequences): 5′-CCCAACTTGTCGAACTTGTCTGACGTCATC-3′.

Transient transfection of the luciferase reporter construct in MCF7-ZEB2 cells and cotransfection with wild-type ZEB2 (pCS3SIP1FS) or ZEB2-DNA binding mutant expression vector (pCS3SIP1NZF3/CZF3-Mut) in parental MCF7 cells were performed using FuGENE HD following the manufacturer’s protocol (Promega). One day after transfection, the cells were treated with doxycycline (1 µg/ml) to induce ZEB2. After 48 h, luciferase activity was measured by using One-GLO luciferase assay according to the manufacturer’s protocol (Promega).

### Epigenetic inhibitors

The cells were treated for 24 h with 1 µM SIRT1 inhibitor (EX-527) and/or 1 µM 5-aza-2′-deoxycytidine (Sigma-Aldrich) along with doxycycline (1 µg/ml). To study the direct effect of the epigenetic inhibitor on gene expression, RNA was extracted after 24 h of treatment. To recover gene expression, the media were replaced with doxycycline-free media for another 24 and 48 h before RNA extraction.

### Quantitative PCR

Total RNA from cells was prepared using the Rneasy mini kit (Qiagen), and cDNA was prepared with SensiFast^TM^ cDNA synthesis kit (Bioline, GC Biotech, Waddinxveen, the Netherlands) following manufacturer’s protocols. Quantitative PCR was performed using SensiFAST^TM^ SYBR no-rox kit following the manufacturer’s protocol in a LightCycler real-time PCR system (Roche Diagnostics, Vilvoorde, Belgium). Primer information is given in Table 1. Each marker was assayed in triplicate in three independent experiments. The expression levels of the genes of interest were normalized to the mRNA level of the HPRT housekeeping gene.

**Table 1:** Primer set for qRT-PCR and ChIP-PCR

Gene	Primer sequences 5′ to 3′
ZEB2	CGAGCGGCATATGGTGACA GCCACACTCTGTGCATTTGAA
RAB25	CTCAGCCCTGGACTCTACCAA TCCGGATGCTGTTCTGTCTCT
CDH1	CGGTTCCGAAGCTGCTAGTC TTGAAGCGATTGCCCCATT
EpCAM	GCGGCTCAGAGAGACTGTG CCAAGCATTTAGACGCCAGTTT
VIM1	GACAATGCGTCTCTGGCACGTCTT TCCTCCGCCTCCTGCAGGTTCTT
SIRT1	TGTGTCATAGGTTAGGTGGTGA AGCCAATTCTTTTTGTGTTCGTG
HPRT	TGACACTGGCAAAACAATGCA GGTCCTTTTCACCAGCAAGCT
ChIP_RAB25_1	ACCTCAGCCTCCCAAAGT TGAGGGCTGAGTGTGCAT
ChIP_RAB25_2	CCCAGCAATGCACACTCA TGGGTGGAGAGATGATGACG
ChIP_RAB25_3	GACACCCAACCTGTCGAACCT CGGAAGCTGAGAACAGGAAGA
ChIP_RAB25_4	TTTGAGAGCTGAGGGTTGAG ATCTTCCTCAGTTCCATTCCC
ChIP_CDH1	GGCCGGCAGGTGAAC GGGCTGGAGTCTGAACTGAC
ChIP_EpCAM	TAGCCTCCACGTTCCTCTATCC TGCTGAGACTTCCTTTTAACCG
ChIP_NC	CACTACGCCTGGCTAATTT TCAGGAGATCGAGACCATC

### Protein extraction and western blotting

Total proteins were extracted with RIPA buffer (50 mM Tris–HCl pH 8, 150 mM NaCl, 1% NP-40, 0.5% sodium deoxycholate and 0.1% SDS) containing Halt^TM^ protease and phosphatase inhibitor cocktail (Thermo Fisher Scientific). Cytoplasmic proteins were extracted in hypo-osmotic buffer (20 mM HEPES pH 7.6, 10 mM KCl, 2 mM MgCl_2_, 1 mM EDTA/EGTA) with 0.1% Tween-20 containing Halt™ protease and phosphatase inhibitor cocktail (Thermo Fisher Scientific) and passed 5 times in a 25G needle. After centrifugation, the supernatant contained cytoplasmic extract, while the nuclear pellet was lysed with RIPA buffer. To extract histones, the nucleus was first purified (1X PBS, 0.5% Triton X-100 and 5 mM sodium butyrate) followed by acid extraction (0.4 M HCl) for 1 h at 4 °C. Acid was neutralized with 2.5× of 1 M Na_2_HPO_4_. For histone marks, 1 µg of histone extract was separated in a 15% gel, and 20 µg of total protein extract was separated on a 10% gel. The following antibodies were used for blotting: H3K27ac (39133), H3K9ac (39917), H3K4me3 (39159), H3K27me3 (39155) (Active Motive), HA (16B12, BioLegend), RAB25 (D4P6P—Cell Signaling Technologies, Leiden, the Netherlands), SIRT1 (07-131, Millipore), HDAC1 (ab7028, Abcam) and beta-tubulin (TUB 2.1) (Sigma-Aldrich).

### Gene expression database analysis

*ZEB2* and *RAB25* expression data were extracted from publicly available datasets from two independent studies on the NCI-60 cell panel (GSE32474 and GSE29288) and from the Cancer Cell Encyclopedia (GSE36133). To study the methylome, we used the NCI-60 Methylome dataset (GSE49143) and focused on the four probes located at or near the promoter region of *RAB25* (cg02448190, cg09243900, cg15896939 and cg19580810). For each dataset, the expression or methylation level of each cell line was compared to the average expression or methylation level of the dataset and log-transformed. Log values were plotted to analyze the correlation between the two parameters.

### Statistical analysis

Statistical analysis was performed using the Graphpad Prism 7.01 software (Graphpad Softwares Inc.). Differences between two samples were analyzed by Student’s *t* test or by ANOVA with selected comparison using Tukey’s post hoc test. Differences were considered significant for *p* values < 0.05. For correlation analysis, Pearson correlation was performed.

## Additional files

**Additional file 1: Figure** **S1.** *ZEB2* and *RAB25* correlation is dependent of the cancer type. Correlation between *ZEB2* (y-axis) and *RAB25* (x-axis) expression from CCLE cell panel datasets analyzed for (**a**, **b**) breast (**c**) colon (**d**) pancreas, (**e**) small-cell lung, (**f**) skin and (**g**) non-small-cell lung cancer cells. Mean of each parameter was calculated, individual cell type values reported to the mean and log2 transformed. Pearson’s correlation test was used to calculate *r* and *p* values.

**Additional file 2: Figure** **S2.** *CDH1* and *EpCAM* are targeted by ZEB2 and SIRT1 modulating H3K9Ac level. (**a**) HA-ZEB2 ChIP assay after induction (+dox) analyzed on *CDH1* and *EpCAM* promoter location using published sequences. (**b**) H3K9Ac ChIP assay performed in MDA-MB-231, 48 h after ZEB2 siRNA treatment (siZEB2). **(c)** *RAB25* mRNA expression measured by qRT-PCR in HT29, MCF7, BT549 and MDA-MB-231. P values were determined using two-way ANOVA (***p < 0.001). (**d**) SIRT1 ChIP and (**e**) H3K9Ac ChIP assay performed after ZEB2 induction (+dox) with SIRT1 inhibitor (EX-527, 1 μM) (+dox/EX-527), in MCF7 analyzed on *CDH1* and *EpCAM* promoter. Enrichments to input were calculated, control values were set as 1 and s.d. is shown. For all analyses, *p* values were determined using two-way ANOVA (*p < 0.05; **p < 0.01). NC = negative control. Three independent experiments were performed for all experiments.

**Additional file 3: Figure** **S3.** DNMT inhibitor 5-aza-2′-deoxycytidine de-repressed *ZEB1*, *SNAI1* and *SNAI2* EMT-TFs which targeted *RAB25* expression. (**a**) *RAB25* and *CDH1* mRNA expression were measured by qRT-PCR 48 h after doxycycline withdrawal (+dox off) with 5-aza-2′-deoxycytidine (1 μM) (+dox/5-aza) and SIRT1 inhibitor (EX-527, 1 μM) (+dox/5-aza/EX-527). *P* values were determined using two-way ANOVA (***p < 0.001). **b** EMT-TFs expressions (*ZEB2*, *ZEB1*, *SNAI1* and *SNAI2*) were measured by qRT-PCR 24 h after 5-aza-2′-deoxycytidine (1 μM) (+ 5-aza) and/or SIRT1 inhibitor (EX-527, 1 μM) treatments (+ EX-527 and +5-aza/EX-527). *P* values were determined using two-way ANOVA (***p < 0.001). (**c**) *RAB25* mRNA expression was measured by qRT-PCR 48 h after *ZEB1*, *SNAI1* and *SNAI2* induction (+dox) in MCF7. *P* values were determined using *t* test (***p < 0.001).

## Abbreviations

5-aza: 5-aza-2′-deoxycytidine
ChIP: chromatin immunoprecipitation
Dox: doxycycline
DNMT: DNA methyl transferase
EMT: epithelial-to-mesenchymal transition
EMT-TF: EMT transcription factor
HA: hemagglutinin
TGF-β: transforming growth factor beta
ZEB: zinc-finger E-box binding homeobox
