# Supplementary figures and images for: ZEB2 stably represses RAB25 expression through epigenetic regulation by SIRT1 and DNMTs during epithelial-to-mesenchymal transition
Source: Epigenetics Chromatin. 2018 Nov 16;11:70. doi: 10.1186/s13072-018-0239-4 (PMC6240308; doi:10.1186/s13072-018-0239-4)

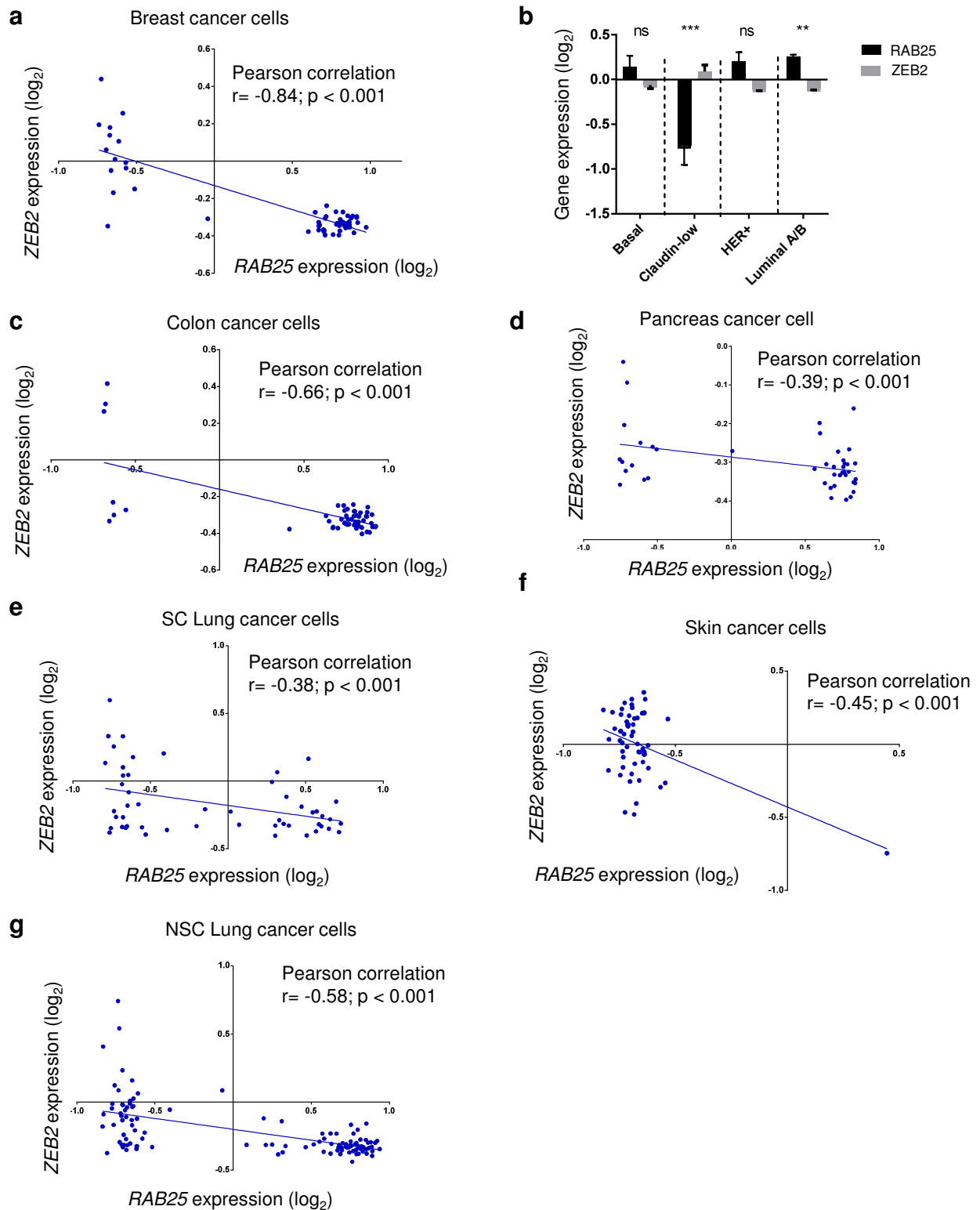

Supplemental Figure 1

Supplement: Supplementary file 1 — Additional file 1: Figure S1. ZEB2 and RAB25 correlation is dependent of the cancer type. Correlation between ZEB2 (y-axis) and RAB25 (x-axis) expression from CCLE cell panel datasets analyzed for (a, b) breast (c) colon (d) pancreas, (e) small-cell lung, (f) skin and (g) non-small-cell lung cancer cells. Mean of each parameter was calculated, individual cell type values reported to the mean and log2 transformed. Pearson’s correlation test was used to calculate r and p values. [file 13072_2018_239_MOESM1_ESM.pdf]

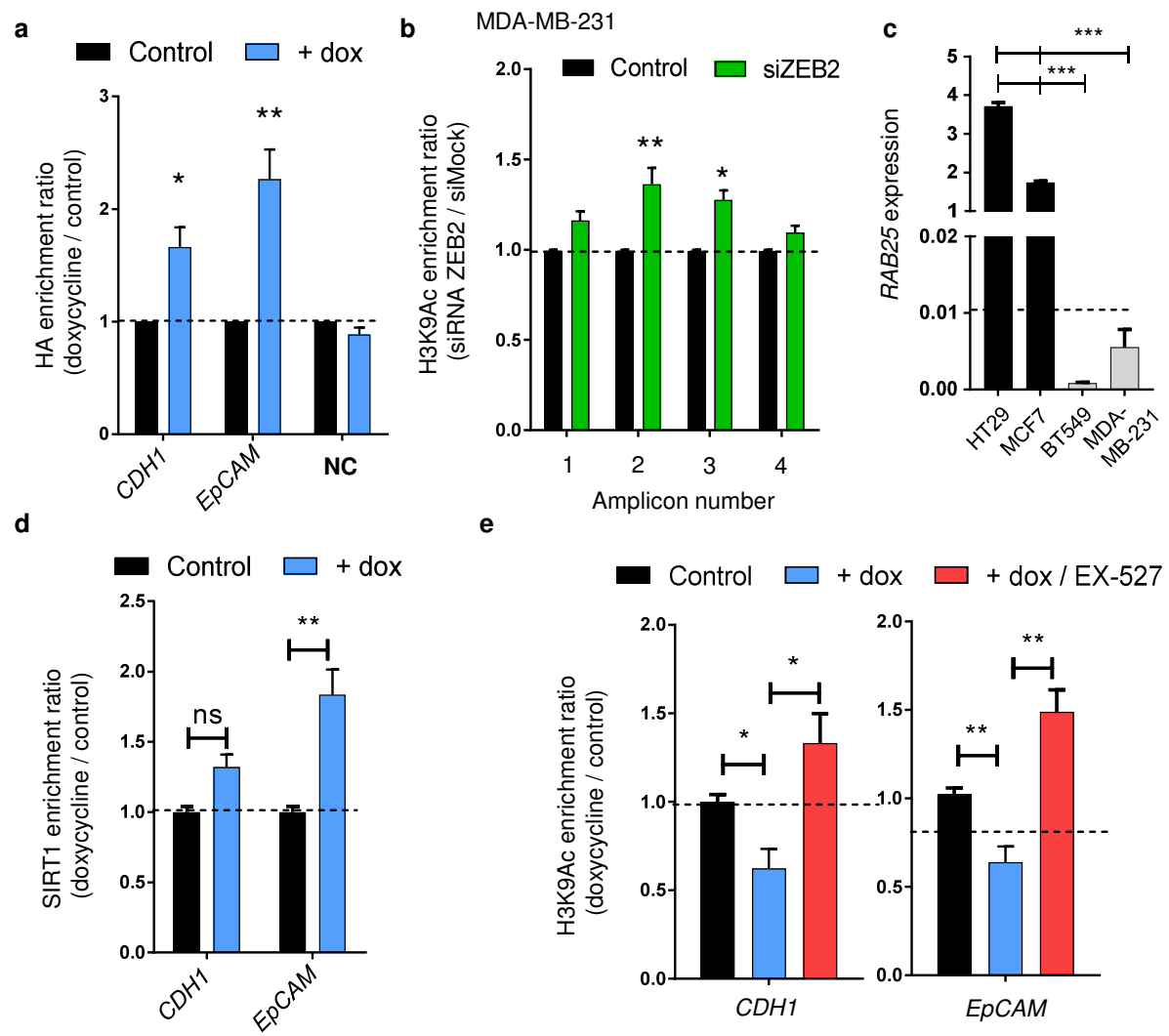

Supplemental Figure 2

Supplement: Supplementary file 2 — Additional file 2: Figure S2. CDH1 and EpCAM are targeted by ZEB2 and SIRT1 modulating H3K9Ac level. (a) HA-ZEB2 ChIP assay after induction (+dox) analyzed on CDH1 and EpCAM promoter location using published sequences. (b) H3K9Ac ChIP assay performed in MDA-MB-231, 48 h after ZEB2 siRNA treatment (siZEB2). (c) RAB25 mRNA expression measured by qRT-PCR in HT29, MCF7, BT549 and MDA-MB-231. P values were determined using two-way ANOVA (***p < 0.001). (d) SIRT1 ChIP and (e) H3K9Ac ChIP assay performed after ZEB2 induction (+dox) with SIRT1 inhibitor (EX-527, 1 μM) (+dox/EX-527), in MCF7 analyzed on CDH1 and EpCAM promoter. Enrichments to input were calculated, control values were set as 1 and s.d. is shown. For all analyses, p values were determined using two-way ANOVA (*p < 0.05; **p < 0.01). NC = negative control. Three independent experiments were performed for all experiments. [file 13072_2018_239_MOESM2_ESM.pdf]
