# Supplementary material for: ZEB2 stably represses RAB25 expression through epigenetic regulation by SIRT1 and DNMTs during epithelial-to-mesenchymal transition
Source: Epigenetics Chromatin. 2018 Nov 16;11:70. doi: 10.1186/s13072-018-0239-4 (PMC6240308; doi:10.1186/s13072-018-0239-4)
Supplement: Supplementary file 3 — Additional file 3: Figure S3. DNMT inhibitor 5-aza-2′-deoxycytidine de-repressed ZEB1, SNAI1 and SNAI2 EMT-TFs which targeted RAB25 expression. (a) RAB25 and CDH1 mRNA expression were measured by qRT-PCR 48 h after doxycycline withdrawal (+dox off) with 5-aza-2′-deoxycytidine (1 μM) (+dox/5-aza) and SIRT1 inhibitor (EX-527, 1 μM) (+dox/5-aza/EX-527). P values were determined using two-way ANOVA (***p < 0.001). b EMT-TFs expressions (ZEB2, ZEB1, SNAI1 and SNAI2) were measured by qRT-PCR 24 h after 5-aza-2′-deoxycytidine (1 μM) (+ 5-aza) and/or SIRT1 inhibitor (EX-527, 1 μM) treatments (+ EX-527 and +5-aza/EX-527). P values were determined using two-way ANOVA (***p < 0.001). (c) RAB25 mRNA expression was measured by qRT-PCR 48 h after ZEB1, SNAI1 and SNAI2 induction (+dox) in MCF7. P values were determined using t test (***p < 0.001). [file 13072_2018_239_MOESM3_ESM.pdf]

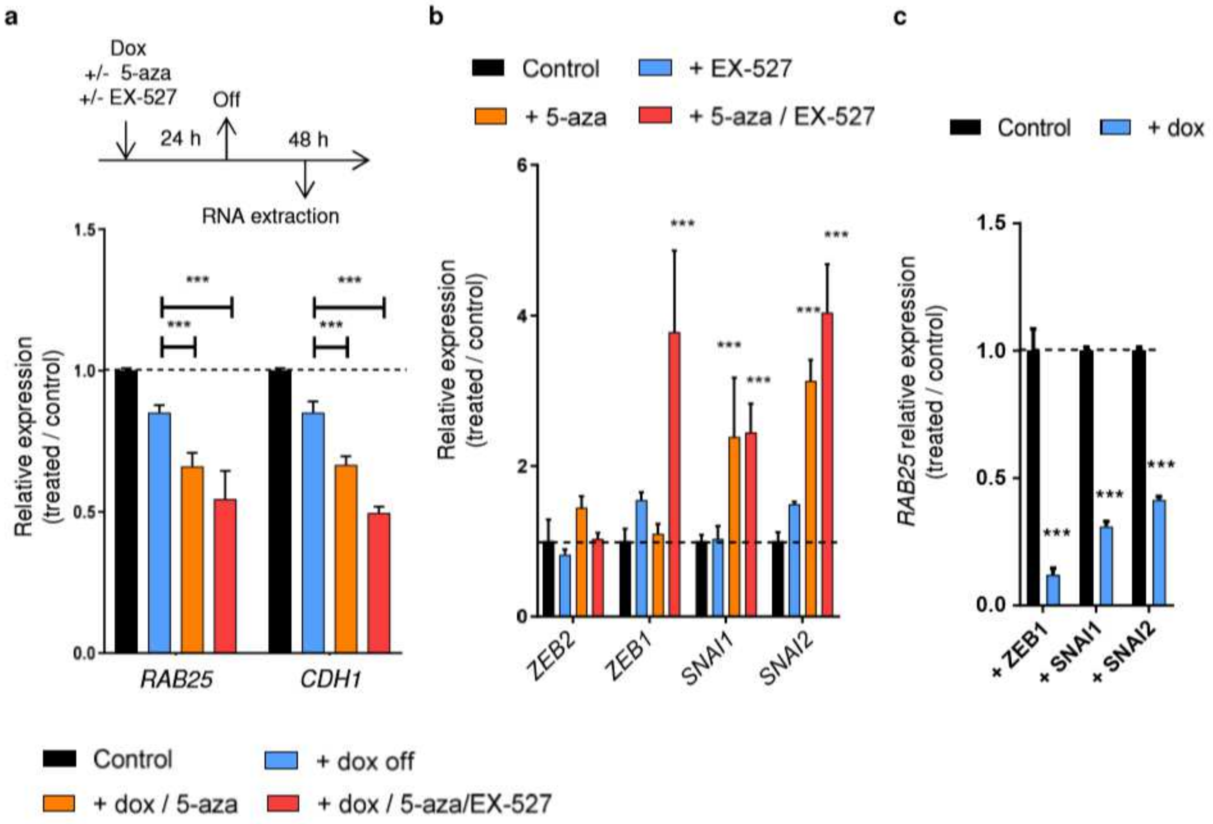

Supplemental Figure 3. DNMT inhibitor 5-aza-2'-deoxycytidine de-repressed *ZEB1*, *SNAI1* and *SNAI2* EMT-TFs which targeted *RAB25* expression. (**a**) *RAB25* and *CDH1* mRNA expression were measured by qRT-PCR 48 hours after doxycycline withdrawal (+ dox off) with 5-aza-2'-deoxycytidine (1µM) (+ dox / 5-aza) and SIRT1 inhibitor (EX-527, 1µM) (+ dox / 5-aza / EX-527). *P*-values were determined using two-way ANOVA (***$p < 0.001$). (**b**) EMT-TFs expression (*ZEB2*, *ZEB1*, *SNAI1* and *SNAI2*) were measured by qRT-PCR 24 hours after 5-aza-2'-deoxycytidine (1µM) (+ 5-aza) and/or SIRT1 inhibitor (EX-527, 1µM) treatments (+ EX-527 and +5-aza / EX-527). *P*-values were determined using two-way ANOVA (***$p < 0.001$). (**c**) *RAB25* mRNA expression was measured by qRT-PCR 48 hours after *ZEB1*, *SNAI1* and *SNAI2* induction (+ dox) in MCF7. *P*-values were determined using t-test (***$p < 0.001$).
